# Long-Term Blockade of Nociceptive Na_v_1.7 Channels Is Analgesic in Rat Models of Knee Arthritis

**DOI:** 10.3390/biom12111571

**Published:** 2022-10-26

**Authors:** Allison R. Reid, Patrice D. Côté, Jason J. McDougall

**Affiliations:** 1 Departments of Pharmacology and Anaesthesia, Pain Management & Perioperative Medicine, Dalhousie University, 5850 College Street, Halifax, NS B3H 4R2, Canada; 2 Department of Biology, Dalhousie University, 1355 Oxford, Halifax, NS B3H 4R2, Canada

**Keywords:** Arthritis, biologics, demyelination, neuropathic pain, voltage-gated sodium channels

## Abstract

The voltage gated sodium channels (Na_v_) 1.7, 1.8, and 1.9 are primarily located on nociceptors where they are involved in signalling neuropathic pain. This study examined the effect of Na_v_1.7 blockade on joint pain using either the small molecule inhibitor PF05089771 or an antibody directed towards the intracellular domain of the ion channel. Male Wistar rats were assigned to one of three experimental groups consisting of either intra-articular injection of 3 mg sodium monoiodoacetate (MIA—joint degeneration group), intra-articular injection of 100 μg lysophosphatidic acid (LPA—joint neuropathy group), or transection of the medial meniscus (MMT—posttraumatic osteoarthritis group). G-ratio calculations were performed to determine potential demyelination and immunohistochemistry was used to measure Na_v_1.7 expression on joint afferent cell bodies. Pain behaviour was evaluated over 3 h by von Frey hair algesiometry and hindlimb weight bearing before and after local administration of PF05089771 (0.1 mg/50 µL). Chronic pain behaviour was assessed over 28 days following peripheral treatment with a Na_v_1.7 antibody (Ab) in conjunction with the transmembrane carrier peptide Pep1. Demyelination and increased Na_v_1.7 channel expression were observed in MIA and LPA rats, but not with MMT. Acute secondary allodynia was diminished by PF05089771 while a single injection of Na_v_1.7 Ab-Pep1 reduced pain up to 28 days. This analgesia only occurred in MIA and LPA animals. Hindlimb incapacitance was not affected by any treatment. These data indicate that joint pain associated with neural demyelination can be alleviated somewhat by Na_v_1.7 channel blockade. Biologics that inactivate Na_v_1.7 channels have the potential to reduce arthritis pain over a protracted period of time.

## 1. Introduction

Chronic pain is a complex phenomenon encompassing nociceptive (due to structural damage), inflammatory, and neuropathic pain phenotypes. The origin of neuropathic pain remains obscure, but likely involves dysregulation of voltage-gated sodium (Na_v_) channels on peripheral neurones [1,2]. These membrane-bound proteins allow the influx of sodium ions into the axon leading to action potential generation and propagation [3]. Following a peripheral neuropathy, the gating mechanism of Na_v_ channels is disrupted such that the neurone fires erratically and neurotransmission becomes defective. On nociceptors, this malfunction produces sharp stabbing sensations which can occur in the absence of any overt damage to the target tissue. There are currently nine known Na_v_ channels (Na_v_1.1−Na_v_1.9) with Na_v_1.7, Na_v_1.8, and Na_v_1.9 being almost exclusively expressed on nociceptors [4]. While systemic administration of non-selective Na_v_ channel blockers such as lidocaine are effective at reducing tissue pain, they can also produce off-target effects such as cardiac arrhythmias which limits their utility as a safe means of analgesia. Inhibitors of Na_v_1.7–Na_v_1.9 therefore offer a more attractive means of treating pain with minimal side-effects.

Pharmacological blockade of Na_v_ channels has been shown to be analgesic in several preclinical and clinical studies. The mechanism of action of these drugs is dependent upon the binding site and conformational state of the Na_v_ channel. Carbamazapine, for example, binds to the α-subunit of the channel and keeps it in the closed, inactivated state such that sodium ions cannot flow across the neurolemma and cause depolarisation [5,6,7]. Other agents such as tetrodotoxin block the channel pore while antibodies directed towards intracellular subunits can alter channel gating properties [8,9]. The arylsulfonamide inhibitor of Na_v_1.7 PF05089771 binds to the extracellular voltage-sensing domain VSD4 of the channel where it blocks action potential generation and reduces afferent firing [10]. Although PF05089771 is highly selective for Na_v_1.7 channels (in humans Na_v_1.7 IC_50_ = 15 nM; Na_v_1.8 IC_50_ = >10,000 nM) [11], the analgesic action of this drug is short-lived. An alternative approach is to use a biologic directed against Na_v_1.7 which has the potential to extend the therapeutic window and improve target selectivity. Antibodies directed towards an external domain have been shown to be effective at modulating ion channel function; however, only a small fraction of the channel protein is available for antibody docking and recognition sites are often shared with other ion channel subtypes [8]. Intracellular domains are more abundant and channel-specific, but the ability for a large molecule such as an antibody to cross the plasma membrane to reach these elements remains a challenge. One approach is to combine the antibody with a carrier peptide which transports these large molecules into the cell [12]. The carrier peptide Pep1 has been shown to block potassium channels in the retina by binding to an intracellular epitope [13]. The current study made use of this technology by combining a Na_v_1.7 antibody with Pep1 to allow selective intracellular blockade of the ion channel to see if this could prolong analgesia compared to a small molecule inhibitor.

Arthritis pain originates in the joint but can lead to central plasticity changes which can make the pain more protracted. It has been postulated that inhibiting this peripheral drive could reverse the central sensitization associated with chronic joint pain [14]. Peripheral sensitization has been observed in the monoiodoacetate (MIA) model of chemically induced osteoarthritis (OA) [15], the medial meniscus transection (MMT) model of post-traumatic OA [16], and the lysophosphatidic acid (LPA) model of joint neuropathic pain [17]. Voltage-gated sodium channels are known to contribute to joint pain and small molecule inhibitors have been found to reduce nociceptor firing locally in the joint [18]. In the LPA model of joint neuropathy, the Na_v_1.8 antagonist A-803467 attenuated nociceptor firing and pain particularly in females [17]. Central administration of the tarantula toxin ProTxII inhibited spinal neuronal hyperexcitability of MIA rats by blocking Na_v_1.7 channels in the dorsal horn [19]. A peripheral role for Na_v_1.7 in modulating arthritis pain has not yet been explored.

The aim of the present investigation was to determine the effect of a small peptide antagonist of Na_v_1.7 (PF05089771) on joint pain using the MIA, LPA, and MMT models. Further experiments using a Na_v_1.7 antibody were also undertaken to validate whether a biologic approach could prolong joint analgesia.

## 2. Materials and Methods

### 2.1. Animals

Male Wistar rats (250–300 g; Charles River Laboratories, Senneville, QC, Canada) were housed in pairs at 22 ± 2 °C on a 12:12 h light:dark cycle (light-on from 7:00 to 19:00). Standard laboratory chow and water were provided ad libitum. All experimental protocols were approved by the Dalhousie University Committee on the Use of Laboratory Animals, which acts in accordance with Animal Research: Reporting of In Vivo Experiments (ARRIVE) and the Canadian Council for Animal Care.

### 2.2. Induction of Arthritis

All arthritis induction procedures were performed under deep general anesthesia (2–4% isoflurane in 100% oxygen at 1 L/min) until animals were unresponsive to toe pinch and corneal blink reflexes. The right knee joint was swabbed with chlorohexidine, ethanol, and betadine. At the conclusion of arthritis induction, animals were closely monitored while recovering under a radiant heat lamp.

To induce OA-like pain, sodium monoiodoacetate (MIA; 3 mg/50 μL dissolved in saline) was injected into the synovial cavity of the right stifle (knee) joint. Animals were tested 14 days later.

Post-traumatic OA was induced by surgical medial meniscus transection (MMT). Here, a small skin incision was made on the medial aspect of the right stifle joint to expose the medial collateral ligament which was then transected in the midsubstance to expose the joint capsule which was similarly cut. The medial meniscus was localised and bisected in the central portion. Successful transection was confirmed by free movement of the meniscus in a medio-lateral plane. The surgical wound was closed with 5–0 polypropylene sutures and the animals were allowed to recover singly housed for several days before being paired with their original cage-mate. Immediately after surgery, animals received a s.c. injection of sustained-release buprenorphine (48–72 h duration). Animals were tested on day 28 following surgery.

Joint neuropathic arthritis was accomplished by an intra-articular (i.artic.) injection of lysophosphatidic acid (LPA; 100 μg/50 μL 5% ethanol in saline). LPA animals were tested 28 days later.

### 2.3. Assessment of Saphenous Nerve Myelination

A small section (approximately 5 mm) of saphenous nerve, proximal to the ipsilateral knee joint, was removed from naïve, MIA, MMT, and LPA rats. Tissues were fixed for several days in 2.5% glutaraldehyde in 0.1 M sodium cacodylate buffer and processed for electron microscopy. Samples were rinsed three times with 0.1 M sodium cacodylate buffer, fixed in 1% osmium tetroxide for 2 h, rinsed in distilled water, placed in 0.25% uranyl acetate overnight at 4 °C, dehydrated via a graduated series of acetone (50%, 70%, 95%, and finally 100%), then embedded in 100% epon araldite resin and cured for 48 h at 60 °C. Thin (100 nm) cross-sections were placed on mesh copper grids and stained with 2% aqueous uranyl acetate for 10 min and rinsed, followed by lead citrate for 4 min before receiving a final rinse. Sections were imaged using a JOEL JEM 1230 transmission electron microscope (JEOL Corp Ltd., Tokyo, Japan) at 2500× magnification. For each sample, three images were captured diagonally across the nerve, and myelin thickness for all imaged fibres (average 98 fibres per nerve) was measured using g-ratio analysis with ImageJ software (Freeware, v1.53, National Institutes of Health, Bethesda, MD, USA). G-ratio was calculated as the square root of the inner axonal area divided by the entire axonal area. Analysed fibres were then subtyped into large diameter (>3 μm) or small diameter (<3 μm) fibres. G-ratio measurements were averaged to give a mean g-ratio for each animal.

### 2.4. Immunohistochemistry

Under isoflurane anaesthesia (2.5–4% in 100% oxygen at 1 L/min), animals (*n* = 4 per group) received an intra-articular injection of the pan-neuronal tracer Fluoro-Gold (2% in saline, 10 μL). Five days later, animals were perfused transcardially with 4% paraformaldehyde and the ipsilateral lumbar dorsal root ganglia (DRG) L3–L5 were harvested. Tissues were post-fixed in 4% paraformaldehyde, cryoprotected through a series of sucrose concentrations, embedded in OCT and sectioned (12 μm thick). Slides were incubated with 10% normal goat serum (NGS) for 1 h at room temperature, washed 3 times with phosphate-buffered saline (PBS) and incubated with guinea pig anti-Nav1.7 primary antibody (1:200 in 1% NGS), at 4 °C overnight. Slides were then washed 5 times with PBS, treated for one hour at room temperature with rabbit anti-guinea pig biotinylated secondary antibody (1:500 in 1% NGS), washed 5 times with PBS and incubated with streptavidin protein Dylight 488 (1:500 in 1% NGS) for 1 h at room temperature. Slides were washed a final 5 times and mounted with fluoromount G. Slides were viewed under a Ziess Axio Imager 2 (Zeiss, Oberkochen, Germany) at 80× magnification at wavelengths of 395 nm for Fluoro-gold and 495 nm for Na_v_1.7 detection. Photomicrographs were taken using an AxioCam HRm camera (Zeiss) and analysed using ZENpro software (Zeiss, Oberkochen, Germany). The percentage of Fluoro-gold positive cells also expressing Na_v_1.7 was calculated for a total of 150 to 200 neurones.

### 2.5. Von Frey Hair Mechanosensitivity

Hind paw mechanosensitivity was assessed using a series of von Frey hair filaments in a modification of the Dixon’s up-down method [20]. Animals were placed in elevated Plexiglas chambers with metal mesh flooring and allowed to acclimate until exploratory behaviour ceased. A von Frey hair was applied perpendicular to the plantar surface of the ipsilateral hind paw with enough force to just slightly bend the hair which was then held in place for three seconds. A positive response (withdrawal, shake or lick of the hind paw) was followed by testing with the next lower strength hair. If there was no response, the next higher strength hair was applied up to a maximum cut-off level of 15 g. After the first difference in response was observed, four more measurements were made and the pattern of responses was converted to a 50% withdrawal threshold calculated using the following formula: 10[Xf + kδ]/10,000; where Xf = value (in log units) of the final von Frey hair used, k = tabular value for the pattern of the last six positive/negative responses, and δ = mean difference (in log units) between stimuli. Animals were returned to their home cages between measurements.

### 2.6. Dynamic Hind Limb Weightbearing

Hind limb incapacitance was assessed using a dynamic weightbearing system (Bioseb, Boulogne, France). Rats were placed in a Perspex chamber (24 cm long × 24 cm wide × 30 cm tall) with a pressure-sensitive floor and a digital camera mounted above. A three-minute video was recorded of the animal moving freely around the chamber. Video footage and sensor pad data were then analyzed to determine weightbearing of each of the limbs. Ipsilateral weightbearing was calculated as a percentage of the total weight placed on both hind paws.

### 2.7. Experimental Timelines

Pain assessments were carried out on day 0 to obtain a baseline (BL) behavioural measurement. On day 14 (MIA) or day 28 (MMT, LPA) von Frey hair algesiometry and dynamic weightbearing were carried out in alert animals at time 0 min to confirm pain development in the models. In separate groups of rats, either the small molecule Na_v_1.7 inhibitor PF05089771 (0.1 mg/50 µL) or vehicle (50 µL 10% DMSO/10% cremophor/80% saline) was then administered subcutaneously (s.c.) over the ipsilateral knee and pain behaviour was assessed over the next 3 h at 30, 60, 120 and 180 min post-injection. To investigate the long-term effects of Na_v_1.7 blockade on joint pain, a single injection of either mouse anti-Na_v_1.7 (25 μg/50 μL in 50 μg PEP-1) or control mouse IgG (25 μg/50 μL in 50 μg Pep1) were administered s.c. over the ipsilateral knee and pain behaviour was assessed every hour for the next 6 h and then over the next four weeks on days 1, 2, 5, 7, 14, 21 and 28 to evaluate chronic effects.

To assess the role of the opioid and endocannabinoid systems in the antinociceptive effect of acute Na_v_1.7 inhibition, either the non-selective opioid antagonist naloxone (1 mg/kg) or the CB_1_-receptor antagonist AM281 (0.5 mg/kg) were administered intraperitoneally (i.p.) 10 min before PF05089771 treatment. Von Frey hair responses were assessed over the next 3 h at 30, 60, 120 and 180 min post-injection.

At the end of each experiment, all rats were euthanized under isoflurane anaesthesia by intra-cardiac injection of euthansol (0.1 mL, 340 mg/mL sodium pentobarbital).

### 2.8. Drugs and Reagents

Normal goat serum and lysophosphatidic acid (LPA) were obtained from Abcam (Toronto, ON, Canada), PF05089771 from Tocris Bioscience (Toronto, ON, Canada), AM281 from Cayman Chemical Company (Ann Arbor, MI, USA), PEP-1 from Abbiotec Inc. (San Diego, CA, USA), Fluoro-gold from Fluorochrome LLC (Denver, CO, USA), mouse IgG from Jackson ImmunoResearch Laboratories, Inc. (West Grove, PA, USA), and guinea pig anti-Na_v_1.7 (for IHC) from Alomone Labs (Jerusalem, Israel).

The mouse anti-Nav1.7 clone N68/6 monoclonal antibody used for behavioural experiments was developed by and obtained from the UC Davis/NIH NeuroMab Facility (Davis, CA, USA). The rabbit anti-guinea pig secondary biotin, streptavidin protein Dylight 488, and fluoromount G were purchased from Thermo Fisher Scientific (Waltham, MA, USA). Cremophor, dimethyl sulphoxide (DMSO), naloxone HCl and sodium monoiodoacetate (MIA) were obtained from Sigma-Aldrich (Oakville, ON, Canada). Isoflurane and euthansol were purchased from CDMV (Dartmouth, NS, Canada).

### 2.9. Statistical Analysis

All data were tested for Gaussian distribution using the D’Agostino & Pearson normality test and expressed as mean ± SEM. Pre- and post- model induction values were compared using a paired Student’s *t*-test. Post-treatment time course data were analysed using a two-way repeated-measures analysis of variance (ANOVA) with Bonferroni’s post-hoc test. G-ratio data were analysed using a one-way ANOVA with Dunnett’s post hoc test. Immunohistochemistry data were analysed by a non-parametric Kruskal–Wallis ANOVA and Dunn’s post hoc test. A *p*-value less than 0.05 was considered statistically significant.

## 3. Results

### 3.1. Changes in Nerve Myelination and Na_v_1.7 Expression

**Figure 1:**
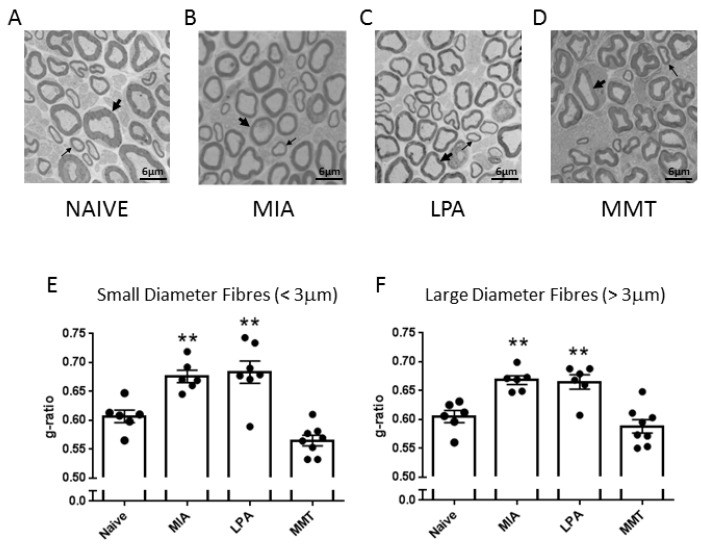
Myelination of Saphenous Nerve Fibres. Representative cross-sectional electron micrographs of ipsilateral saphenous nerves harvested from (**A**) naïve, (**B**) MIA, (**C**) LPA, and (**D**) MMT rats. Myelin thickness (g-ratio analysis) was significantly reduced in MIA- and LPA-treated joints in both small diameter (**E**) and large diameter (**F**) neurones. Myelin thickness was not affected by MMT surgery at the 28 day timepoint. Thick arrows signify large diameter axons; thin arrows refer to small diameter axons. ** *p* < 0.01 compared to naive, one-way ANOVA with Dunnett’s post-hoc test. Data are expressed as mean ± S.E.M (*n* = 6–8 animals/group).

In the MIA and LPA models of arthritis, ipsilateral saphenous nerve fibres showed significant demyelination of both small (Figure 1E) and large diameter (Figure 1F) axons compared to naïve control animals (*p* < 0.01 one-way ANOVA). Day 28 MMT animals, on the other hand, showed no difference in myelin thickness with either small diameter (*p* = 0.08) or large diameter fibres (*p* = 0.55). An average of 98 fibres were counted per nerve for 6–8 animals per treatment group.

**Figure 2:**
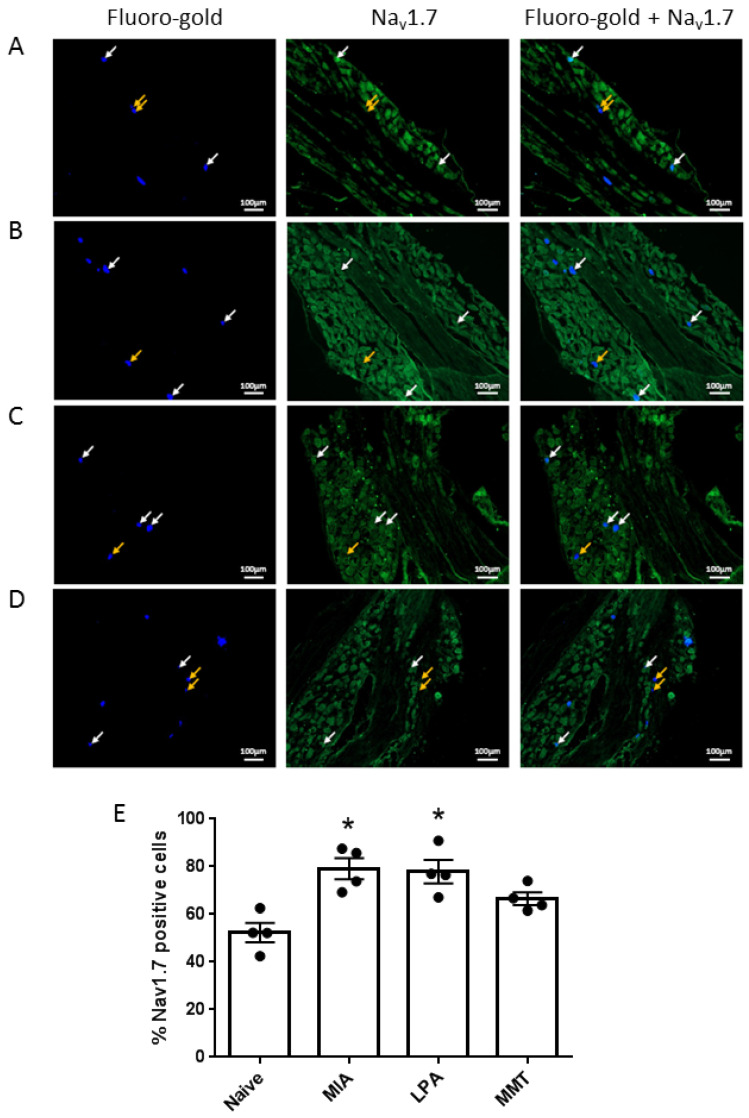
Expression of Nav1.7 in Dorsal Root Ganglia. Immunofluorescent micrographs showing Fluoro-gold-traced joint afferents (blue) and Na_v_1.7 labelled cells (green) in (**A**) naïve, (**B**) MIA, (**C**) LPA, and (**D**) MMT rats. Yellow arrows indicate joint afferents that are negative for Na_v_1.7 and white arrows point to Fluoro-gold positive joint afferents that co-express Na_v_1.7. The percentage of joint neuronal cell bodies that express Na_v_1.7 was increased in MIA and LPA, but not MMT, rats compared to naive control animals (**E**). * *p* < 0.05 compared to naïve control, Kruskal–Wallis ANOVA with Dunn’s post hoc test. Data are expressed as mean ± S.E.M (*n* = 4 animals/group).

Immunohistochemical examination of dorsal root ganglia, showed an increase in Na_v_1.7 expression in the neurones innervating the ipsilateral knee joint of MIA and LPA rats (*p* < 0.05, Figure 2E). The MMT surgery model did not induce a significant change in Na_v_1.7 expression (*p* = 0.6, Figure 2E).

### 3.2. Effect of Acute Na_v_1.7 Channel Blockade on Pain Behaviour

**Figure 3:**
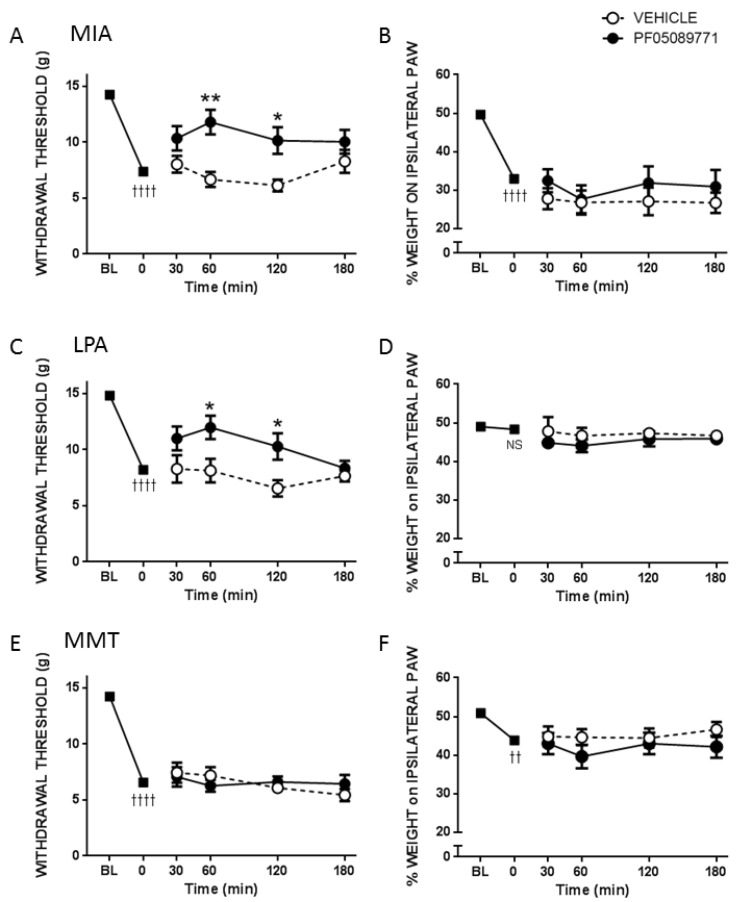
Effect of PF05089771 on Joint Pain. Compared to vehicle, acute local administration of PF05089771 (0.1 mg in 50 μL) reduced von Frey hair mechanosensitivity in MIA (**A**) and LPA (**C**) animals, but not in the surgical MMT model (**E**). Peripheral blockade of Na_v_1.7 had no effect on hindlimb weight bearing in any model tested (**B**,**D**,**F**). †† *p* < 0.01, †††† *p* < 0.0001, two-tailed paired Student *t*-test between baseline (BL) vs. t = 0 min; * *p* < 0.05, ** *p* < 0.01, two-way RMANOVA with Bonferroni’s post hoc test. Data are expressed as mean ± S.E.M. (*n* = 7–13 animals/group).

Fourteen days after intra-articular injection, MIA produced a dramatic decrease (47.7 ± 3.8%) in the force required to illicit a withdrawal response in the ipsilateral hindpaw (*p* < 0.0001, *n* = 11–13 animals/group, Figure 3A) and a marked drop (36.8 ± 3.4%) in the amount of weight borne on the ipsilateral hindlimb (*p* < 0.0001, Figure 3B). Twenty-eight days after treatment of the knee with LPA, there was an observable drop (44.7 ± 3.8%) in withdrawal thresholds (*p* < 0.0001, *n* = 7–8 animals/group, Figure 3C) but no significant change (1.1 ± 2.6%) in hindlimb weight bearing (*p* = 0.4, Figure 3D). Surgical destabilisation of the joint by MMT surgery also produced a significant drop (53.1 ± 2.9%) in withdrawal threshold (*p* < 0.0001, *n* = 11–13 animals/group, Figure 3E) but only a modest decrease (13.2 ± 3.9%) in weight bearing (*p* < 0.01, Figure 3F).

The small molecule inhibitor of Na_v_1.7, PF05089771, reversed the secondary allodynia observed in MIA and LPA treated knees (*p* < 0.01, two factor repeated measures ANOVA, Figure 3A,C), but not in the MMT model (*p* = 0.9, Figure 3E). Conversely, PF05089771 had no effect on hindlimb weightbearing in any of the three models tested (*p* > 0.05, Figure 3B,D,F).

### 3.3. Role of the Endogenous Opioid and Endocannabinoid Systems in PF05089771 Responses

**Figure 4:**
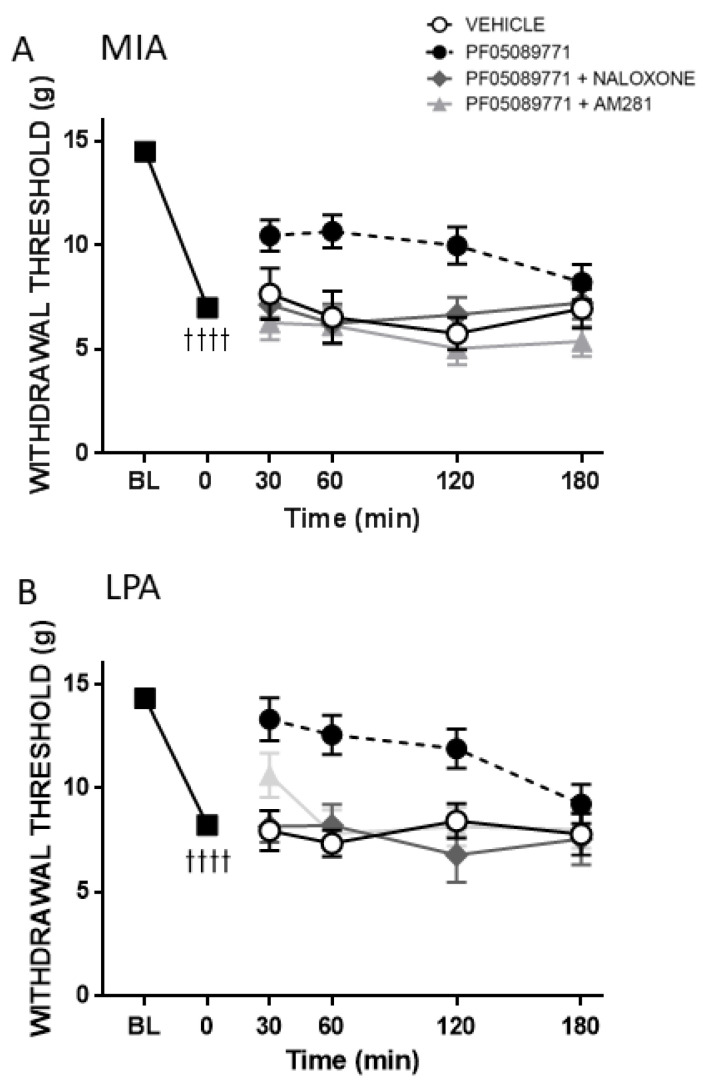
Involvement of the Endogenous Opioid and Endocannabinoid Systems in PF05089771 Responses. Blockade of endogenous opioid receptors with naloxone or CB_1_ receptors with AM281 significantly reduced the anti-allodynic effect of PF05089771 in MIA- (**A**) and LPA- (**B**) treated rats. *p* < 0.05, two-way RMANOVA; †††† *p* < 0.0001, two-tailed paired Student *t*-test between baseline (BL) vs. t = 0 min. Data are expressed as mean ± S.E.M (*n* = 8–16 animals/group).

To determine the involvement of endogenous opioids and endocannabinoids in acute Na_v_1.7 blockade, rats were pre-treated with either the non-selective opioid receptor antagonist naloxone or the CB_1_ receptor antagonist AM281. Naloxone completely blocked the anti-allodynic effect of PF05089771 in the MIA and LPA models (*p* < 0.05, *n* = 8–16 animals/group, Figure 4A,B). Local treatment with AM281 also inhibited the effect of PF05089771 in both the MIA and LPA models (*p* < 0.05, *n* = 8–16 animals/group, Figure 4A,B). Neither naloxone nor AM281 had any effect on mechanosensitivity when given alone (data not shown). Naloxone and AM281 were not assessed in the dynamic weightbearing test or the MMT model because PF05089771 had no effect on this parameter.

### 3.4. Prolonged Blockade of Na_v_1.7 by Selective Antibody Treatment

**Figure 5:**
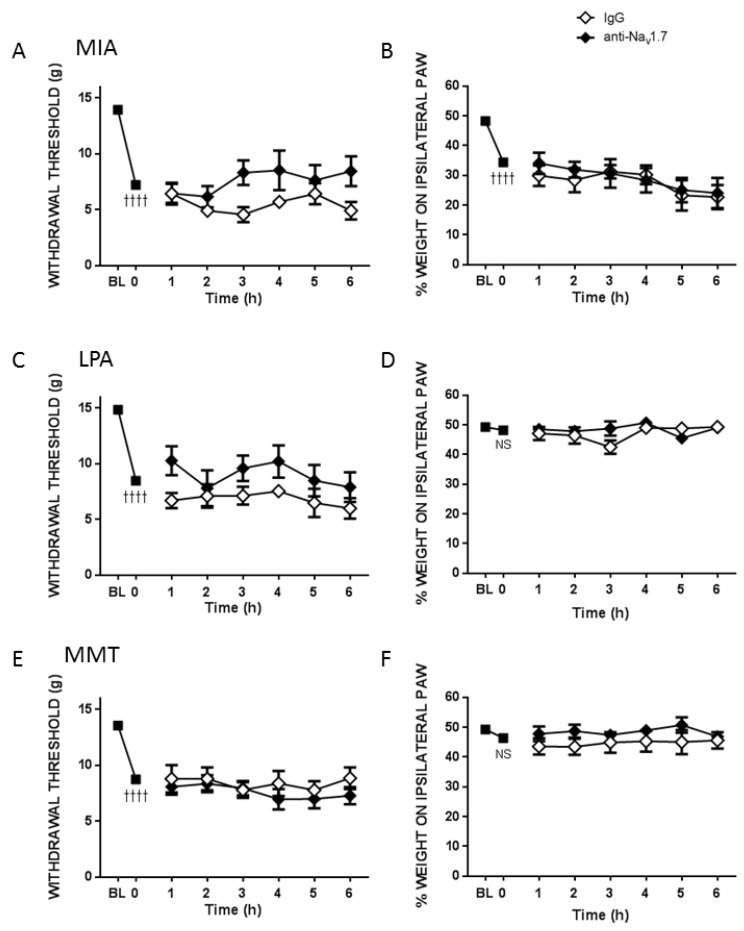
Acute Effect of anti-Na_v_1.7 Treatment on Joint Pain. Local, subcutaneous injection of anti-Na_v_1.7 (25 μg in 50 μL) over the knees of MIA (**A**,**B**), LPA (**C**,**D**), and MMT (**E**,**F**) animals had no effect on hindpaw mechanosensitivity nor hindlimb weight bearing over the acute 6 h time-course compared to IgG controls, *p* > 0.05, two–way RMANOVA. †††† *p* < 0.0001, paired Student *t*-test of baseline (BL) vs. t = 0 min. Data are expressed as mean ± S.E.M (*n* = 8–9 animals/group).

**Figure 6:**
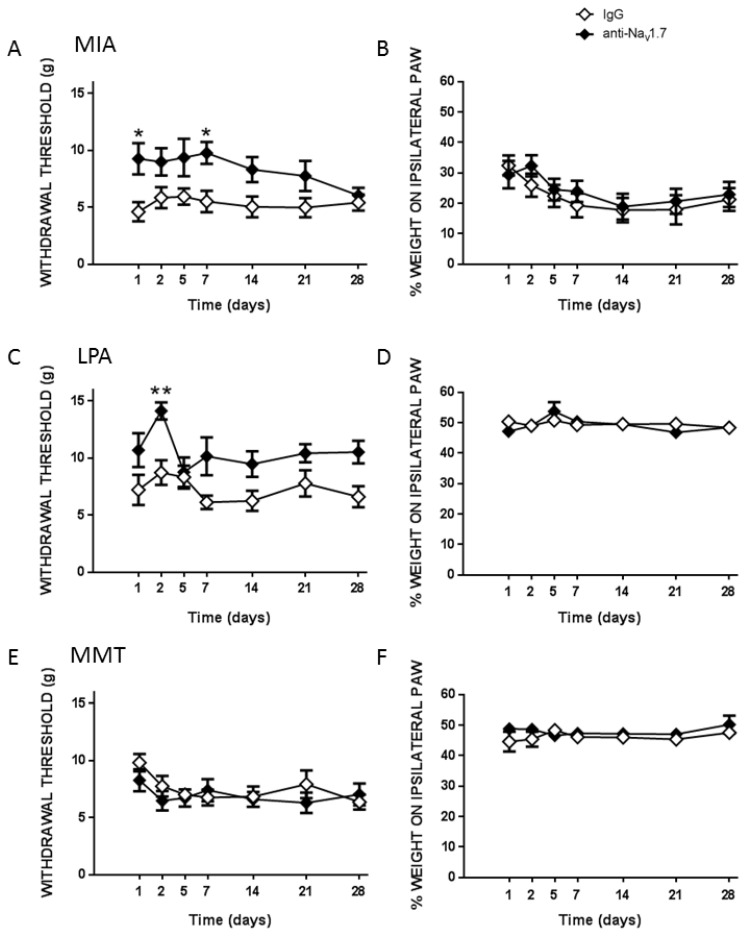
Chronic Effect of anti-Na_v_1.7 Treatment on Joint Pain. Following a single, local injection of anti-Na_v_1.7 (25 μg in 50 μL), hindpaw mechanosensitivity was significantly reduced over 28 days in MIA (**A**) and LPA (**C**) animals, but not MMT rats (**E**). Prolonged blockade of Na_v_1.7 had no effect on hindlimb weight bearing over the 28-day time-course (**B**,**D**,**F**). * *p* < 0.05, ** *p* < 0.01, two-way RMANOVA with Bonferroni’s post hoc test. Data are expressed as mean ± S.E.M (*n* = 8–9 animals/group).

The effects of a single dose of the Na_v_1.7 antibody were analysed for acute responses (1 to 6 h post-treatment) and chronically (1 to 28 days post-treatment). In the acute phase, the Na_v_1.7 antibody had no effect on hindpaw mechanosensitivity in any of the experimental models compared to the IgG control antibody (*p* > 0.05, *n* = 8–9 animals/group, Figure 5A,C,E). Antibody blockade of neuronal Na_v_1.7 channels had no effect on weightbearing in any of the three models during this acute phase (*p* > 0.05, Figure 5B,D,F). Monitoring MIA and LPA animals over the subsequent 28 days, however, revealed that pain was significantly attenuated which lasted throughout these extended timepoints (*p* < 0.01, Figure 6A,C). Antibody treatment had no effect on pain in the MMT model (*p* = 0.5, Figure 6E) nor on hindlimb weight bearing in any model (*p* > 0.05, Figure 6B,D,F).

## 4. Discussion

An emerging feature of the various arthritides is that joint disease can culminate in sensory nerve damage leading to the development of neuropathic pain. While the mechanisms responsible for peripheral neuropathy continue to be determined in arthritis, Na_v_ channels are believed to be intrinsically linked with generating joint neuropathic pain. The subcategorization of Na_v_ channels led to the identification of Na_v_1.7, Na_v_1.8, and Na_v_1.9 as being almost exclusively expressed on the neurolemma of nociceptors making them an attractive target for the control of pain generation and neurotransmission. In the realm of joint pain, Na_v_1.8 blockade was effective at reducing nociceptor activity particularly in females [17,18]. A limitation of these studies was that a small peptide inhibitor was used which restricts analgesia to a matter of hours. In the present investigation, the role of Na_v_1.7 in joint pain using three distinct arthritis models was assessed. Comparisons were made between acute inhibition of Na_v_1.7 using the small molecule PF05089771 and long-term blockade with an antibody approach. Further mechanistic studies were performed to investigate the involvement of the endocannabinoid and endogenous opioid systems following acute Na_v_1.7 channel block.

To investigate peripheral neuropathy, saphenous nerve g-ratios were measured in the MIA, MMT, and LPA models of joint damage. Demyelination was observed in both small (<3 µm) and large (>3 µm) diameter axons associated with MIA and LPA-treated knees, whereas myelin thickness was unaltered in MMT animals. These findings are consistent with previous studies which showed a decrease in myelin thickness in the MIA and LPA models [21,22] while myelination was unaffected by MMT surgery [16]. Levels of Na_v_1.7 were also elevated in the cell bodies of neurones innervating the joints of MIA and LPA rats, but not in animals that had undergone MMT surgery. These data align with what has previously been reported in the Freund’s complete adjuvant model of chronic inflammatory joint disease where Na_v_1.7–1.9 were upregulated on joint nociceptors with concomitant heightened pain behaviour [23]. The increased expression of Na_v_1.7 and the loss of axonal myelin in MIA and LPA rats described here suggest that these models would be more predisposed to Na_v_1.7 channel blockade than MMT rats. Indeed, the small molecule inhibitor PF05089771 reduced secondary allodynia in MIA and LPA joints whereas MMT animals showed no response to Na_v_1.7 inhibition. Local administration of PF05089771 had no effect on hindlimb weightbearing in any of the models tested. The reason for this may be that dynamic weightbearing is not sensitive enough to detect a meaningful analgesic response to the drug or that the pain processing mechanisms are different between von Frey hair-evoked nociception versus non-evoked gait changes in these models. Evidence supporting this concept comes from a study where weightbearing deficits in a femoral bone cancer pain model occurred independently of peripheral Na_v_1.7 channels [24]. Thus, Na_v_1.7 channels do not appear to contribute to hindlimb incapacitance but do play a role in the development of other pain modalities.

Pain processing and sensitivity are known to be different between the sexes with females being more likely to experience pain symptoms [25]. With respect to nociceptor-dominant sodium channels, female arthritic rats demonstrate heightened mechanical pain and are more responsive to Na_v_1.8 channel blockade than males [17]. In isolated mouse DRG neurones, PF05089771 was more effective at blocking sodium currents in females indicating a sex difference in Na_v_1.7 ion channel function [26]. The present study only used male rats and therefore these pain experiments should be repeated in female arthritic animals to uncover any potential sex differences.

In addition to a direct effect on action potential generation and propagation, Na_v_1.7 channel gating may also be regulated by endogenous opioids and endocannabinoids. Individuals with a loss of function mutation to Na_v_1.7 channels are congenitally insensitive to pain. Pain can be awakened in these individuals, however, by infusion of naloxone suggesting a functional link between Na_v_1.7 channels and the endogenous opioid system [27]. Using multiple rodent pain models, Chen et al. discovered that the analgesic properties of Na_v_1.7 channel blockers derived from tarantula venom could be reversed by co-administration of naloxone [28]. With respect to the endocannabinoid system, anandamide can reduce sodium currents flowing through Na_v_1.7 channels and it is thought that this mechanism contributes to the analgesic properties of this endocannabinoid [29]. Another endocannabinoid, 2-arachidonylglycerol, can also reduce neuronal sodium flux in response to lipopolysaccharide-induced inflammation [30]. It is believed that endocannabinoids, and possibly opioids, gain access to the Na_v_ channel pore via fenestrations located in the lipid bilayer leading to channel blockade [31,32]. In the present study, both naloxone and AM281 attenuated the analgesic effect of PF05089771 indicating that the endogenous opioid and endocannabinoid systems are involved in joint Na_v_1.7 inhibition. Future molecular studies are required to elucidate the intricate crosstalk between opioid and cannabinoid receptors and Na_v_1.7 channel gating properties on joint nociceptors.

The main limitation of using small molecules to block Na_v_ channels is that their therapeutic window is in the order of minutes and therefore not suitable for the treatment of chronic pain states. The chemical properties of peptide inhibitors are such that their poor oral bioavailability and rapid tissue clearance precludes long-lasting analgesia. Specially engineered antibodies tagged with Na_v_1.7 channel blocking toxins have been shown to inhibit channel activity for days using only nanomolar concentrations [33,34]. Antibodies that are directed towards extracellular domains are fraught with reduced specificity and accessible binding sites are limited. Since the majority of the Na_v_ channel occurs in the cytoplasm and the associated subunits are highly conserved, antibodies that target intracellular components offer a more desirable means of selectively modulating the ion channel [8]. The principal challenge with this approach is that antibodies are not able to pass through cellular membranes to reach intracellular components. To circumvent this drawback, the carrier protein Pep1 has been used to transport large antibodies into cells to modulate ion channel activity [13]. A single, local injection of a Na_v_1.7 channel selective antibody in conjunction with Pep1 reduced MIA and LPA-induced pain for up to 28 days but was ineffective during the initial 6 h. These data provide compelling evidence that a biological approach to Na_v_1.7 channel blockade can produce long-term analgesia to arthritis pain. The lack of effect of the Na_v_1.7 antibody in the MMT model, however, suggests that this approach may not be suitable for all types of joint pain. Further experiments are required to determine the underlying factors responsible for this divergence in antibody modulation of Na_v_1.7 ion channels. A further limitation of this study was that the Pep1-Na_v_1.7 Ab treatment was not tested against other sodium channel subtypes. Therefore, further experiments are required to determine antibody specificity as well as identify the intracellular epitope to which the antibody is binding.

## 5. Conclusions

In summary, joint disease in which there is demyelination of articular nerves causes an upregulation of nociceptor Na_v_1.7 channel expression and this translates into heightened pain sensation. Joint pain may be controlled acutely using small molecule blockers such as PF05089771 and this effect is regulated by endogenous opioids and endocannabinoids. Antibodies directed towards intracellular domains of Na_v_1.7 can silence the channel leading to long-term analgesia. Future studies are required to identify the type of joint pain that can be alleviated by Na_v_1.7 channel blockers; however, they remain an interesting target for the clinical management of arthritis pain.

## Data Availability

Data are available from the corresponding author upon request.
